# A Screen-Peck Task for Investigating Cognitive Bias in Laying Hens

**DOI:** 10.1371/journal.pone.0158222

**Published:** 2016-07-13

**Authors:** Amanda Deakin, William J. Browne, James J. L. Hodge, Elizabeth S. Paul, Michael Mendl

**Affiliations:** 1 School of Veterinary Science, University of Bristol, Langford, United Kingdom; 2 Centre for Multilevel Modelling, University of Bristol, Bristol, United Kingdom; 3 School of Physiology, Pharmacology and Neuroscience, University of Bristol, Bristol, United Kingdom; University of British Columbia, CANADA

## Abstract

Affect-induced cognitive judgement biases occur in both humans and animals. Animals in a more negative affective state tend to interpret ambiguous cues more negatively than animals in a more positive state and vice versa. Investigating animals’ responses to ambiguous cues can therefore be used as a proxy measure of affective state. We investigated laying hens’ responses to ambiguous stimuli using a novel cognitive bias task. In the ‘screen-peck’ task, hens were trained to peck a high/low saturation orange circle presented on a computer screen (positive cue–P) to obtain a mealworm reward, and to not peck when the oppositely saturated orange circle was presented (negative cue–N) to avoid a one second air puff. Ambiguous cues were orange circles of intermediate saturation between the P and N cue (near-positive–NP; middle–M; near-negative–NN), and were unrewarded. Cue pecking showed a clear generalisation curve from P through NP, M, NN to N suggesting that hens were able to associate colour saturation with reward or punishment, and could discriminate between stimuli that were more or less similar to learnt cues. Across six test sessions, there was no evidence for extinction of pecking responses to ambiguous cues. We manipulated affective state by changing temperature during testing to either ~20°C or ~29°C in a repeated measures cross-over design. Hens have been shown to prefer temperatures in the higher range and hence we assumed that exposure to the higher temperature would induce a relatively positive affective state. Hens tested under warmer conditions were significantly more likely to peck the M probe than those tested at cooler temperatures suggesting that increased temperature in the ranges tested here may have some positive effect on hens, inducing a positive cognitive bias.

## Introduction

Concern for animal welfare is based on the assumption that animals are able to experience negative (and positive) emotional (affective) states. Emotions can be operationally defined as ‘states elicited by rewards and punishers’ (rewards inducing positive states and punishers inducing negative states), where a reward is something that an animal will learn to work for and a punisher is something that it will learn to avoid [1]. We cannot know for certain whether these affective states include a conscious subjective component, but we can develop proxy measures for such states, particularly those that reliably reflect affective valence (whether an animal is in a positive or negative state), the key determinant of welfare [2].

In humans, the phenomenon of affect-induced cognitive bias, whereby people in a negative emotional state pay more attention to threatening stimuli, are more likely to recall negative memories, and judge ambiguous stimuli more negatively (are relatively more ‘pessimistic’), represents a ‘cognitive indicator’ that appears to reliably reflect affective valence [2], [3]. Animal studies now indicate that biases in judgements of ambiguity may also follow this pattern, suggesting that judgement bias may be a useful indicator of animal affective valence and hence welfare.

The first published study of cognitive bias in animals [4] investigated the effect of unpredictable housing conditions on rats’ responses to a lever press task, finding that rats housed in unpredictable conditions had a diminished expectation of reward when tested under ambiguity. The study provided a generic paradigm for testing judgement biases that involves training animals to make one response (*P*: e.g. lever press) to a cue (e.g. a tone of a particular frequency) predicting a positive outcome (e.g. food reward), and a different response (*N*: e.g. no lever press) to a different cue (e.g. a different tone) predicting a negative outcome (e.g. white noise). Once the discrimination is learned, ‘ambiguous’ cues (e.g. tones of intermediate frequency) are occasionally presented to see whether the animal makes response *P*, an ‘optimistic’ judgement of ambiguity putatively associated with positive affect, or response *N*, a ‘pessimistic’ judgement. This paradigm has been implemented in a variety of forms in many studies of cognitive bias across a range of species, mainly mammals [3], [5], [6], [7], [8].

The current study focuses on the development of a cognitive bias assay to investigate welfare in a highly important commercial animal, the laying hen, 6.7 billion of which were farmed worldwide in 2012 [9]. Although many measures of hen welfare exist, their relationship to affective state is often unclear. For example, commonly used indicators of poor welfare such as elevated corticosterone concentration and decreased physical condition did not change in the predicted directions in preferred (peat flooring, a perch and a nest box) or non-preferred (wire floor with no perch or nest box) environments which, following our operational definition, should induce relatively positive and negative affective states respectively [10]. New measures of welfare are therefore still needed, and cognitive bias is one measure that has the potential to inform us about affective valence in this species.

Studying cognitive bias in hens is also relevant from a comparative neuropsychological point of view, to determine whether cognition-emotion relationships are similar in mammalian and bird species. To date, affect-induced cognitive biases have been demonstrated in a passerine species, the starling [11], [12], [13], whilst the only other bird species to have been studied, the chicken, has produced equivocal findings.

Wichman *et al*. [14] trained laying hens on a spatial cognitive bias task. Birds were rewarded with food for moving to a bowl on trials in which it was placed in one location in a test arena, but not rewarded for approaching it on trials when it was in another location. Birds in basic pens did not differ significantly in cognitive bias (their responses to the bowl when placed in ambiguous intermediate locations), or in measures of anticipatory behaviour compared to birds in enriched pens. The manipulations in housing may not have been sufficient to induce a change in affective state. In the task used, chickens required an average of 150 trials to achieve the discrimination learning criterion (moving to the rewarded location at least 5s faster than they moved to the unrewarded location), and 10 out of 37 birds did not reach criterion.

A similar spatial judgement bias task was used by Seehuus *et al*. to study chicks [15]. Birds were trained to discriminate between locations that predicted either a rewarding mealworm or an aversive quinine-soaked piece of puffed rice. Some differences in latencies to approach ambiguous cues were seen between chicks temporarily deprived of a foraging substrate compared to when not deprived, but not when access to a feeding area or dark area was restricted. Chicks achieved the discrimination criterion of a 2s difference in running to the rewarded and non-rewarded locations after 110 trials spread over 13 days.

Hernandez *et al*. [16] used a more complicated task based on a paradigm used in starlings by Brilot *et al*. [13] in which laying hens were exposed to two boxes of the same shade of grey on each trial, one on the right and one on the left. The birds were trained that, for example, when the boxes were both light grey a small reward (1 mealworm) would be available from the left box and nothing from the right box, but when they were both dark grey a large reward (4 mealworms) would be available from the right box and nothing from the left box. Once the discrimination had been learnt, Hernandez *et al*. investigated whether birds made left (‘pessimistic’) or right (‘optimistic’) choices when presented with an ambiguous shade of grey, and how this was affected by a 5-minute period of isolation to induce an anxiety-like state. Hens took 23 days (c.185 trials) to learn the initial discrimination, and ten out of the original 30 hens did not achieve the criterion of 9 out of 10 correct choices on 2 consecutive days. The affect manipulation did not generate a judgement bias in this study.

Two further studies of chicks used a runway task to test male chicks’ willingness to approach either a mirror or an image of an owl, or morphed chicken/owl images [17], [18]. These stimuli seemed to work well for male chicks, and an advantage of this task is that extensive training is not required. However, perhaps due to this, clear generalisation curves of responses to the ambiguous cues were not so apparent, and mirror reflections or pictures of conspecifics may work less well as positive stimuli for adult hens who generally choose to avoid unknown individuals [19], [20].

Given the potential for using cognitive bias tests to assess poultry affect and welfare, our aim in this study was to design a new cognitive bias task for adult laying hens that would be relatively quick to train and which most birds would be able to learn. The task should yield clear generalisation curves across ambiguous cues and be sensitive to affect manipulations. As in previous tests of starlings [11], [12], [13] and hens [16], we used visual stimuli as the discriminative cues and designed stimuli which engage the chickens’ propensity to peck at small objects such as grains of food. Because chickens possess highly developed colour vision and have been found to be more attracted to colours than greys [21], we used coloured stimuli.

In our screen-peck task, hens were trained to peck a high (or low) saturation orange cue presented on a computer screen to receive a food reward and refrain from pecking a low (or high) saturation orange cue in order to avoid a negative air puff. Variation in saturation was used because birds are good at making this type of discrimination [22]. Using both reward and punishment in this task increases the affective impact of decision outcomes which, in turn, may render decisions more sensitive to affective state. It also offers the possibility of assessing differences in both reward anticipation (which may be more closely linked to depressive-like states) and punishment anticipation (which may be more closely linked to anxiety-like states) [23], although other factors may influence which ambiguous cues reveal a biased decision [3].

Once the task was trained, we investigated whether repeated testing using non-reinforced ambiguous probe cues of intermediate colour saturation resulted in extinction of responding to these cues as has been found in other studies e.g. [24]. We then investigated the effect of a temperature-based affect manipulation on decision-making under ambiguity. Following the logic that exposure to a preferred stimulus or event induces a relatively positive affective state [1], we reasoned that a preferred temperature would induce positive affect. Abeyesinghe *et al*. [25] found that broiler chickens chose a 40°C environment significantly more often than a 15–20°C environment, and this is concordant with the finding that the thermo-neutral zone of domestic fowl may be as high as 32.2–37.7°C [26], in keeping with the tropical ancestry of these birds. We therefore imposed warmer and cooler temperatures during the ambiguity tests.

## Materials and Methods

### Ethics statement

All work was approved by our Institutional Animal Care and Use Committee (University of Bristol Animal Welfare and Ethical Review Body) under UB/12/024. We also conducted the study in compliance with ASAB ethical guidelines. The hens were rehomed to small responsible free-range holdings after the study.

### Animals, housing and experimental apparatus

**Fig 1:**
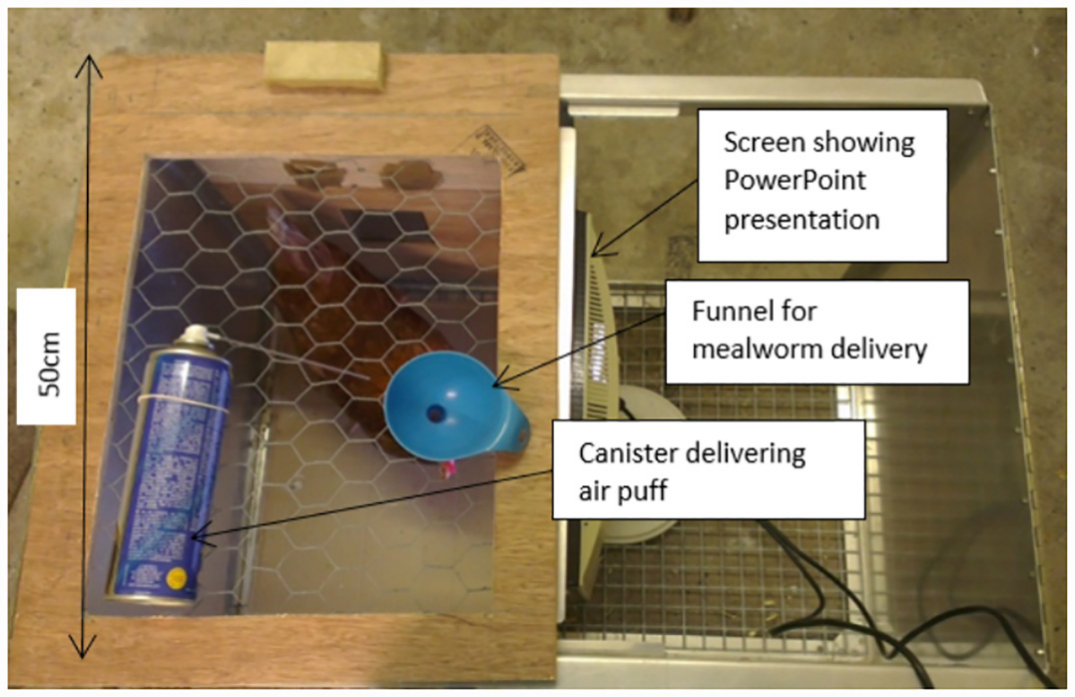
Test apparatus. Birds are positioned in the test box; one side of which is made of clear Perspex enabling the bird to see a computer screen that presents the cues. A correct peck response to a P cue is rewarded by a mealworm delivered through a funnel from above the bird by the experimenter whereas an incorrect, no-peck, response is not rewarded. An incorrect peck response to an N cue is punished by a 1 second air puff delivered from behind the bird, whereas a correct, no-peck, response is not punished.

A total of eight 18 week-old Shaver Brown laying hens were used in this study. The hens were housed in 298 x 348cm pens with sawdust litter, three perches, 10 nest boxes and had layer pellets, water and grit available *ad libitum*. They were kept on a 12 hour light-dark cycle (light period 07:00–19:00 hours) and ambient temperature ranged from 19–21°C. Experiments took place in a 116 x 116cm training pen in a separate room. Birds were trained in a 50 x 50cm wooden box within this pen. One side of the box was made of clear Perspex allowing the bird to see a computer screen (27 x 33cm) on which an experimenter-controlled Microsoft PowerPoint presentation showing the test stimuli was displayed (Fig 1). The lid of the box was made of wire mesh allowing the experimenter to drop mealworms through a funnel to reward the bird for a correct peck response to a P cue, or to administer a one second puff of inert compressed air (Sprayduster, AF International, UK) if the bird made an incorrect peck response to an N cue. Trials were recorded with a video camera (Canon Legria HF R506) positioned outside of the box with an aerial view of the bird and computer screen.

### Training and testing

#### Shaping phase

On day one, hens were trained individually in the training pen to associate mealworm delivery with a click sound from a dog-training clicker (Clix multi-clicker). This involved clicking and rewarding the hen as it wandered around the training pen. After three 5 minute sessions all of the eight hens appeared to be anticipating the mealworm reward after the click by orientating to the experimenter with an alert stance. On day two, hens were individually placed in the test box with the computer screen presenting a blank white display and were further trained to associate the clicker with mealworm reward (2 x 10 minute sessions per bird). On day three, a 1 x 1cm black dot was displayed in the centre of the screen and hens were only rewarded when they orientated towards the dot or pecked the dot or a nearby location on the screen. When hens were reliably pecking the black dot to receive a reward over the course of two consecutive 10 minute sessions they moved on to positive cue training. For those hens that were not reliably pecking the dot after 10 training sessions, the dot was made to shrink and grow in size in an attempt to direct their attention towards it.

#### Positive cue (P) training phase

For trials in this phase of training, a smaller (0.5cm diameter) black dot was displayed at the centre of the screen, but this was now surrounded by a large orange-scale circle (3.5cm diameter). This was the P cue. We chose to use an orange saturation scale because chickens do not appear to have an inherent preference for or against this colour [21], [27], [28]. In addition, orange was not present in the home environment minimising the chance of induced preferences that can sometimes result from exposure to coloured feeders or drinkers [29]. Birds were trained to peck at the P cue in order to receive a mealworm reward. For half the birds (N = 4), the P cue was a high saturation orange colour (HS group; MS PowerPoint HSL scale–H = 19; S = 250; L = 119) and for the other half it was a low saturation orange colour (LS group; MS PowerPoint HSL scale–H = 19; S = 50; L = 119). When a bird successfully pecked at the P cue, they received a click and a mealworm reward and the screen went white for a 5-second inter-trial-interval (ITI) before reappearance of the P cue. If a bird did not peck the stimulus during a 10 second period, the screen went blank for the ITI and then the circle reappeared. When birds pecked the P cue in 90% of presentations to receive a reward over three consecutive 10 minute sessions (two sessions per day), they moved on to discrimination training.

#### Discrimination training phase

In this phase, birds were presented with a sequence of P and N cues. The N cue was of the opposite saturation (high/low) to the P cue and 40 cues were presented in total per session (two sessions per day). A pseudorandom sequence of presentations was generated (Microsoft Excel) and modified to ensure that the first and last presentation was a P cue and there were no more than three consecutive N cue presentations by swapping N cues at these points to P cues to avoid this. This meant that in most cases, birds were shown slightly more presentations of the P cue than the N cue. Birds were clicked and rewarded for pecking at a P cue and were given a one second air puff for pecking an N cue. Reinforcement rate remained at 100% for the duration of the task. Birds were given 10 seconds to decide whether or not to peck the screen, and a 5 second ITI (blank white screen) between presentations. In order to move on to the ambiguous probe test, birds had to achieve a success rate of >90% on both N and P cue presentations over three consecutive discrimination sessions.

#### Ambiguous cue testing phase

In this phase, three ambiguous probe (unrewarded) cues were interspersed between the N and P cue presentations. Ambiguous probe cues were intermediate between the training cues (100, 150 and 200 saturation on the orange-scale). For each bird, one ambiguous probe was ‘central’ between the P and N (150; middle—M), one was central between M and P (100 or 200 depending on contingency; near positive: NP), and one was central between M and N (200 or 100 depending on contingency; near-negative—NN). Each test session comprised 40 stimuli presentations. Each ambiguous probe cue was presented twice per session (six ambiguous cue presentations; 17 P cue presentations; 17 N cue presentations). The order in which the ambiguous probe cues were presented was the same for all birds but differed over three test sessions so that all birds saw three sequences (session 1: M, NP, NN; NP, M, NN; session 2: NN, M, NP; M, NN, NP; session 3: NP, NN, M; NN, NP, M). The exact cue sequence in a session was randomised (MS Excel rand() function), and then adjusted so that there were no more than three consecutive presentations of an N cue by swapping N cues for P cues where necessary. Ambiguous probe cues were never presented consecutively in an attempt to avoid birds becoming frustrated with the lack of reward after such cue presentation. All three sessions were repeated a second time so each bird completed a total of six ambiguous cue test sessions. On each trial in each session, birds had 10 seconds to decide whether or not to peck the cue. Pecking P and N cues resulted in a mealworm or an air puff respectively. If an ambiguous probe cue was pecked, the screen immediately went white for five seconds before the next presentation. If a cue (P, N or ambiguous probe) was not pecked, the screen went white for five seconds after the 10 seconds choice period. Responses to ambiguous probe cues were not reinforced. Whether or not cues were pecked and the latencies to peck them, were recorded for each trial from the video recording.

#### Affective state manipulation phase

Following ambiguous cue testing, birds were given once-weekly ‘refresher’ discrimination training sessions for two weeks and then subjected to a second set of ambiguous cue tests in which an affective state manipulation was applied. Variation in temperature was used as there is evidence that chickens show clear temperature preferences (see Introduction).

The ambiguous cue testing sessions were exactly the same as previously except that a 250W red heat lamp was positioned above the testing box. The lamp was turned on shortly before testing under ‘Hot’ conditions to heat up the box and left on whilst hens were tested. Under ‘Cold’ conditions the lamp was switched off and instead a red light bulb was illuminated to control for any effects of red light *per se*. Temperature was recorded shortly before each session with a digital thermometer and varied between 19.6–21.8°C in the ‘Cold’ condition (mean temperature = 20.45 ± 0.20 (SEM)) and 28–30.7°C in the ‘Hot’ condition (mean temperature = 29.14 ± 0.20). A repeated-measures design was implemented so that each hen had three ambiguous cue test sessions in the Hot condition and three in the Cold condition. Hot and cold conditions alternated and birds had a normal discrimination session between each so that birds completed an ambiguous probe session every other day. Three of the birds in each group (HS group and LS group) started with a cold trial whilst the other two started with a hot trial. Proportion of cues pecked and latency to peck during the 10-second decision period of each trial were recorded as previously.

### Statistical analysis

Analysis was carried out using IBM SPSS Statistics 21. For each cue type, the mean proportion of trials on which it was pecked during the 10s presentation time, and the total latency (to peck) over the series of trials (with each no peck trial scored as 10s) was calculated for each session. To calculate an average measure, the total latency was divided by the number of trials in which a peck actually occurred as this deals with the censoring of the no peck observations (and is equivalent to assuming an exponential distribution for individual trials). For example, suppose a bird was presented with cue one on eight trials of a session and did not peck it by 10s on three of these trials, but did peck it on the other five trials with latencies of: 2s, 3s, 5s, 7s, 8s, then the total peck latency time for cue one would be 10+10+10+2+3+5+7+8 = 55s but, because only five trials involved pecking, the average latency would be calculated as 55s/5 = 11s. For the ambiguous cue tests, means for each cue across all six sessions were calculated apart from for the first screen-peck task (no affect manipulation) in which data for P and N latencies were only available for the last three sessions for technical reasons (percentage pecked data was available for all six sessions). Studentized residuals were checked for normality, sphericity, and homogeneity of variance. Where assumptions were satisfied, repeated-measures General Linear Models were used to analyse data. A binary logistic regression with whether or not a probe was pecked (0/1) as the dependent variable and session, hen and probe as categorical covariates was used to investigate whether peck responses to the ambiguous cues changed across sessions as previous studies have suggested that subjects may extinguish their responses to repeated presentations of unrewarded probes e.g. [24]. When data could not be transformed to fulfil assumptions, non-parametric tests were used. Data are presented as means with a standard error of the mean (SEM) as a measure of variance.

## Results

### Shaping, positive cue, and discrimination training phases

After clicker training over two days, birds were exposed to the black on-screen training dot. Four out of the eight birds were reliably pecking the dot to receive a reward after five sessions and were moved on to the positive cue-training task. The other four stayed on the training dot for 10 further sessions before the dot was made to grow and shrink in an attempt to grab their attention. One further bird began to peck at the dot at this stage whilst the other three birds did not and were removed from the study. All five remaining birds were ready to move on to the discrimination training phase after four positive cue training sessions. In this phase, the cue predicting mealworm reward (P) was of the high saturation orange colour whilst the cue predicting air puff (N) was of the low saturation colour for three birds and the opposite was the case for the other two birds. The birds took an average of 8.20 ± 0.49 sessions to move on to the ambiguous cue-testing phase.

### Ambiguous cue testing phase

**Fig 2:**
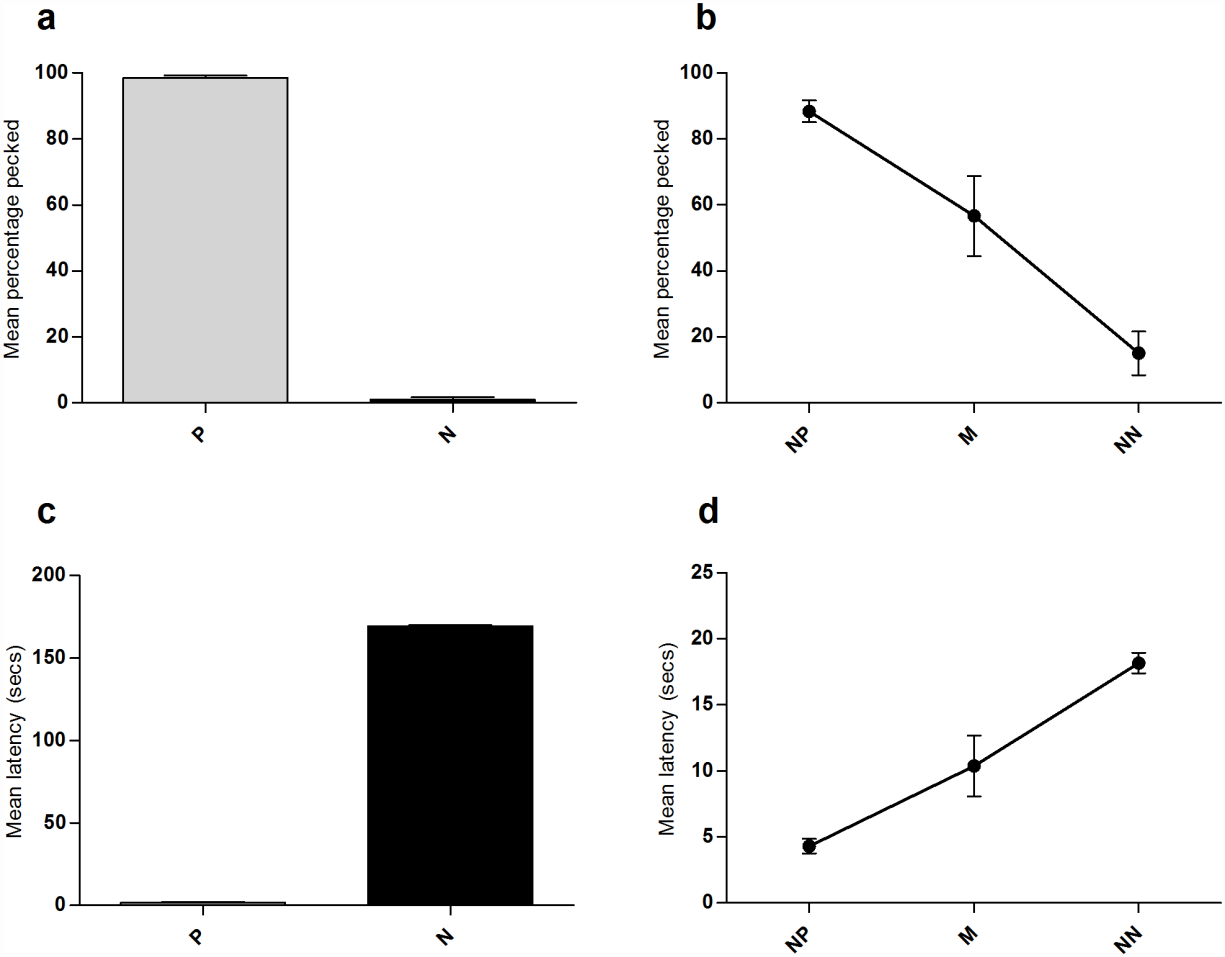
Screen-peck task peck percentage and latency data collected during the 6 ambiguous probe sessions. Birds saw 17 presentations of each of the P and N cues, and 2 of each probe (near-positive; NP, Middle; M, Near-negative; NN) per session. (a) Mean percentage of positive (P) and negative (N) cues pecked. (b) Mean percentage of ambiguous probe cues pecked. (c) Mean latency to peck positive (P) and negative (N) cues. (d) Mean latency to peck ambiguous probe cues. Error bars show ± 1 SEM.

Hens pecked the P cue significantly more frequently than the N cue (Wilcoxon signed rank Z = -2.03, P = 0.042) indicating that they had learnt the discrimination between these two cues (Fig 2a). There was a strong effect of cue type on responses in the ambiguous cue tests (Friedman test: Χ^2^_2_ = 9.58, P = 0.008) with birds being most likely to peck the NP cue, followed by the M then NN cues (Fig 2b). Latency data reflected the same findings; hens pecked P cues significantly quicker than N cues (Wilcoxon signed rank Z = -2.02, P = 0.043; Fig 2c). Response latencies to probe cues increased from NP to M then NN (F_2,8_ = 27.29, P<0.001; Fig 2d).

**Fig 3:**
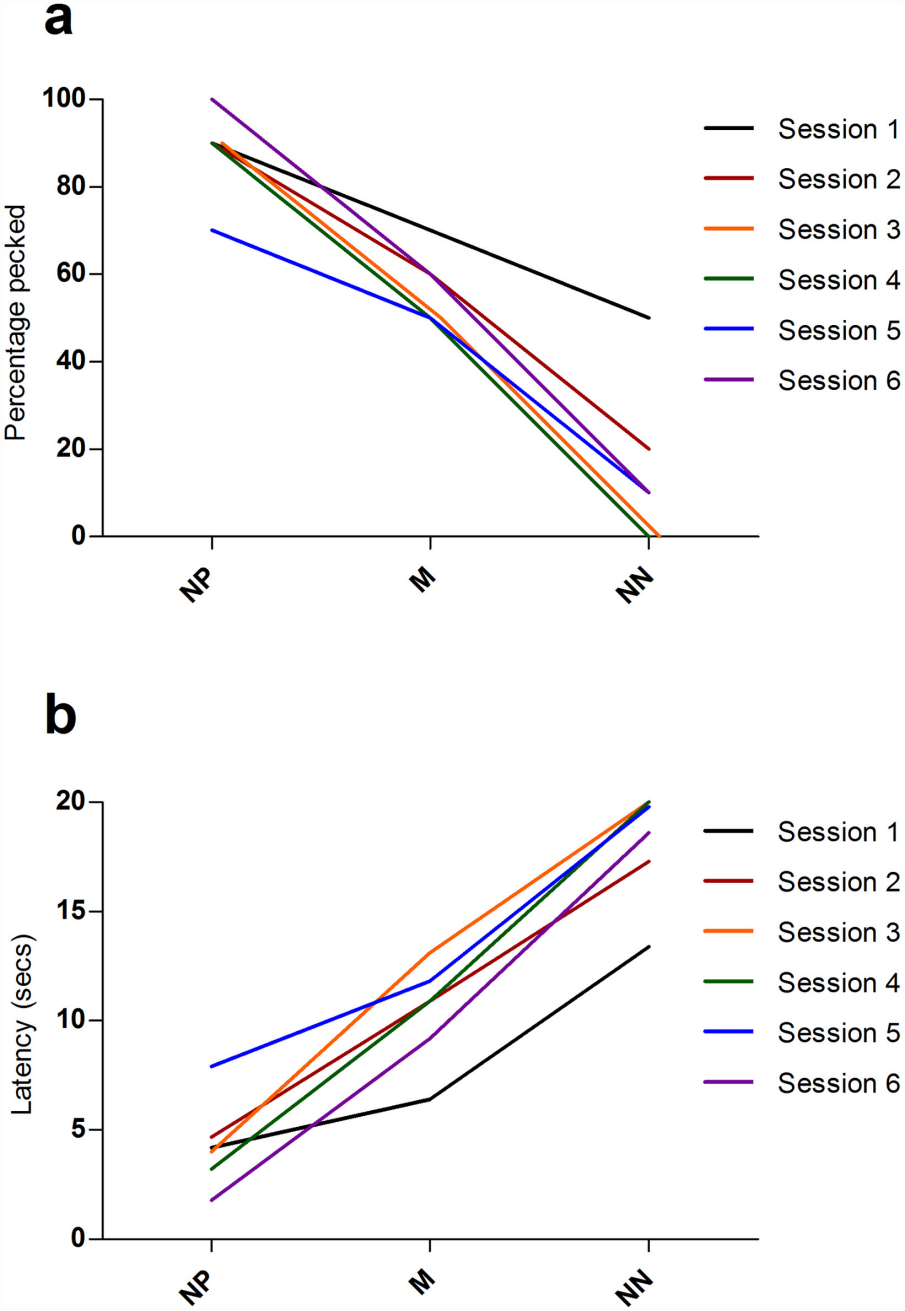
Pattern of responses to ambiguous probe cues across the 6 test sessions in the screen-peck task. (a) Average percentage of times each probe cue was pecked across the 6 test sessions. (b) Average latency (seconds) to peck each probe cue across the 6 test sessions. Each line represents one test session.

To investigate whether there was any evidence of extinction of pecking responses to repeated presentations of unrewarded probe cues, we analysed summary data from the six separate ambiguous cue test sessions. There was no effect of session number on the number of probe cues pecked (binary logistic regression: Χ ^2^_5_ = 5.89, P = 0.317) indicating no evidence of extinction of the peck response to ambiguous cues across session (Fig 3a). Session number also had no significant effect on the latency to peck cues (Friedman test: Χ ^2^_5_ = 10.60, P = 0.060; Fig 3b).

### Affective state manipulation phase

**Fig 4:**
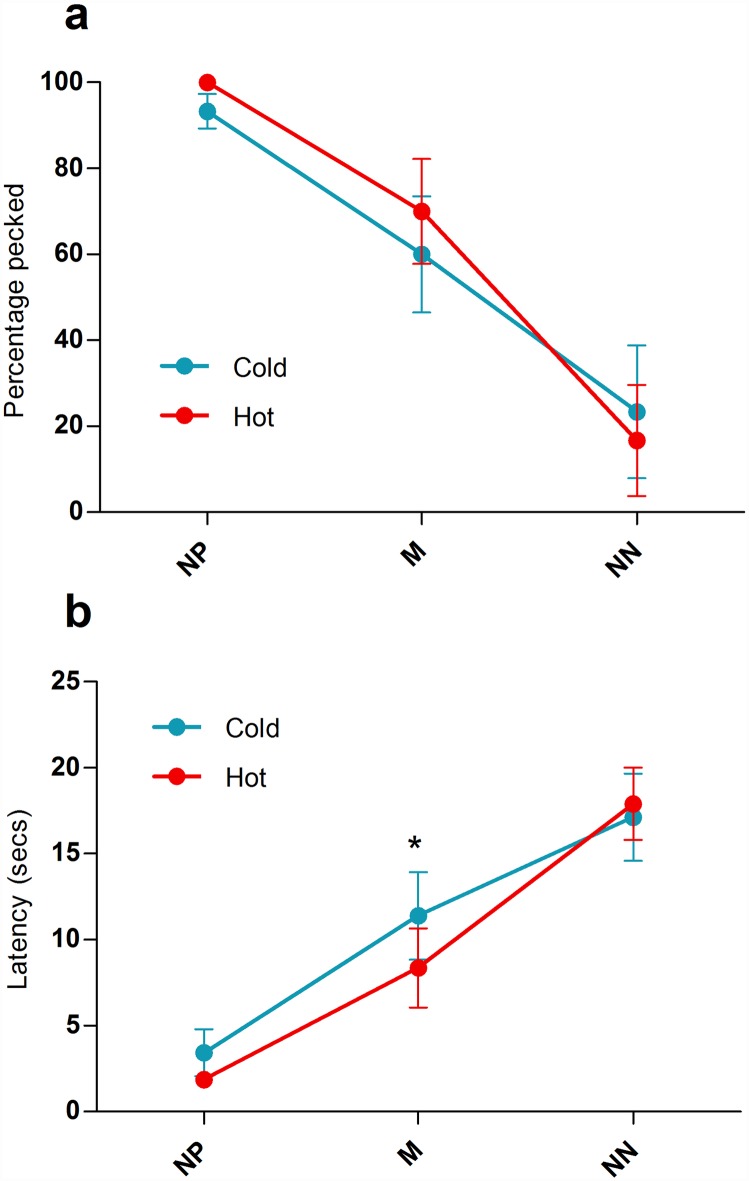
Pattern of responses to ambiguous probe cues in the affective state manipulation phase. Each subject was tested under cold conditions 3 times and under hot conditions 3 times. (a) Average percentage of stimuli pecked in the cold (blue line) compared to the hot (red line) condition for the 5 subjects tested. (b) Average latencies to peck stimuli in the cold (blue line) compared to the hot (red line) condition for the 5 subjects tested. Error bars show ± 1 SEM; * = p<0.05.

As in the preceding phase, hens continued to prefer to peck the P cue to the N cue in both the Cold and Hot conditions (mean percentage of cues pecked: Cold—P = 97.65 ± 2.35, N = 1.96 ± 1.07, Wilcoxon signed rank Z = -2.03, P = 0.042; Hot–P = 96.08 ± 3.45, N = 1.18 ± 0.78, Wilcoxon signed rank Z = -2.03, P = 0.042) but there were no significant differences between the two conditions in the percentage of P and N cues pecked (P cue: Wilcoxon signed rank Z = -1.34, P = 0.180; N cue: Wilcoxon signed rank Z = -0.55, P = 0.581). Probe cue type had a strong effect on the probability of pecking in both conditions (Cold: Friedman test Χ^2^_2_ = 9.58, P = 0.008; Hot: Friedman test Χ ^2^_2_ = 9.33, P = 0.009) showing the same pattern of change across cues as in the previous ambiguous cue tests (Fig 4a). Temperature did not significantly affect the percentage of probe cues pecked (NP: Wilcoxon signed rank Z = -1.41, P = 0.157; M: Z = -1.73, P = 0.083; NN: Z = -1.34, P = 0.180).

Latency data indicated that hens pecked the P cue significantly faster than the N cue in both conditions (mean latency to peck cues (seconds): Cold—P = 1.43 ± 0.12, N = 163.43 ± 5.91, Wilcoxon signed rank Z = -2.02, P = 0.043; Hot—P = 1.86 ± 0.39, N = 168.20 ± 1.08, t_4_ = 117.44, P<0.001) but there were no significant differences between the two conditions in the time to peck P and N cues (P cue: t_4_ = 1.49, P = 0.212; N cue: Wilcoxon signed rank Z = -0.37, P = 0.715). Probe cue type affected latency to peck in the same way as in the previous ambiguous cue tests (mean latency to peck cues (seconds): Cold: Friedman test Χ ^2^_2_ = 10.00, P = 0.007; Hot: Friedman test Χ ^2^_2_ = 10.00, P = 0.007). Increased temperature significantly decreased the latency to peck M cues but not NP cues or NN cues (NP: Wilcoxon signed rank Z = -1.10, P = 0.273; M: t_4_ = 3.86, P = 0.018; NN: Wilcoxon signed rank Z = -1.63, P = 0.102; Fig 4b).

## Discussion

In the screen-peck task, hens were trained to peck at a coloured circle (positive cue, P) to obtain a mealworm reward and to not peck at a differently coloured circle (negative cue, N) to avoid an air puff. Within the time available, five out of eight birds successfully learnt to peck at the screen and discriminate between the two coloured circles. Three were eliminated due to failure to peck the screen at all rather than failure to discriminate between colours, indicating a 100% success rate once birds acquired the necessary operant response. Training on this task (including clicker training) took a maximum of 29 sessions (average 25 sessions with each session comprising of 40 trials) over a total of 16 days (average 10.6 days). In terms of days, this was comparable to or faster than most other chicken judgement bias tasks (see Introduction). However, the number of trials required to achieve criterion (c.1000) was much higher than for other tasks. Nevertheless, this would be offset by the potential to completely automate the task using a computerised Skinner box touchscreen apparatus.

The ambiguous cue-testing phase was carried out over six days during which hens’ responses to three ambiguous colour cues were investigated. Hens produced clear generalisation curves, pecking at the probes with a decreased likelihood and an increased latency as they became more similar to the N cue. We also considered how session number influenced the percentage of probe cues pecked and the latency to peck probe cues, as there is some evidence that animals may learn that probe cues are unrewarded, decreasing the likelihood that they will treat them as a positive cue e.g. [24]. In this study, hens did not extinguish pecking responses to probe cues or take significantly longer to peck probe cues over sessions 1–6 suggesting that this method has potential to be used as a repeated measure of affective state over time.

The influence of affect was investigated by testing hens in either a Hot (29.14°C ± 0.20) or Cold condition (20.45°C ± 0.20) in a repeated measures cross-over design. There is evidence to suggest that hens prefer warmer temperatures [25], [26] and therefore it was expected that hens in the Hot condition would be in a more positive affective state (as elicited by the experience of a rewarding stimulus) [1] and hence more likely to peck ambiguous probe cues than birds in the Cold condition, reflecting a more ‘optimistic’ cognitive bias.

In line with our hypothesis, hens in the Hot condition pecked M cues significantly faster than hens in the Cold condition. However, the temperature manipulation had no significant effect on the latency to peck NP or NN cues. This could be due to more uncertainty around the M cue, as evidenced from the larger variance in response between birds (Fig 2b and 2d), and it is likely that the influence of background affective state on decision-making is most pronounced under conditions of high uncertainty/ambiguity where limited current information is available to guide choice [23].

There is some evidence, in humans at least, that a higher body temperature may lead to faster reaction times potentially via increased psychomotor vigilance [30], [31]. However, the possibility that birds responded quicker to M probes because the increased temperature led to a quicker pecking response is unlikely as reactions to reference (P and N) cues and other ambiguous cues were not significantly affected.

The temperature manipulation had no significant effect on the percentage of probe cues that were pecked. It is possible that the influence of a short-term temperature change was not extreme enough to affect the actual propensity to peck probe cues but did affect the time taken to make a decision. A more long-term temperature manipulation; for example altering the temperature of hens’ home pens throughout the experimental period and testing them in the same conditions might lead to more pronounced differences in responses. On the other hand, if even small, temporary temperature alterations are capable of altering hens’ affective state, this has implications for hen welfare in situations where temperature fluctuates or is consistently cooler or warmer than the desired range. Here, the ‘optimistic’ cognitive bias associated with an increased temperature indicates the potential for enhancing welfare by increasing positive affect as well as by decreasing negative affect [32], [33].

In conclusion, the screen-peck task offers promise as a method for assessing affect-induced judgement biases in adult hens. It is relatively quick to train, offers opportunities for automation, yields clear generalisation curves which are repeatable across time, and appears to be able to detect the influence of an affect manipulation. This task is also easily adaptable for use in other species including mammals e.g. using a nose poke or paw reach instead of a pecking response. We are currently using the screen-peck task to assess the influence of long-term housing treatments on bird welfare.
